# Data for iTRAQ-based quantification of the cardiac proteome of rainbow trout (*Oncorhynchus mykiss*) at rest and with exercise training

**DOI:** 10.1016/j.dib.2017.05.016

**Published:** 2017-05-10

**Authors:** L.A. Dindia, S.L. Alderman, T.E. Gillis

**Affiliations:** Department of Integrative Biology, University of Guelph, Guelph, Ontario, Canada

## Abstract

This data article presents the first description of the rainbow trout cardiac ventricle at the level of the proteome, with more than 700 proteins identified and quantified using isobaric tags for relative and absolute quantitation (iTRAQ) and LC-MS/MS. The abundances of these proteins were compared across 4 durations of moderate exercise training (0, 4, 7, and 14 d), and a total of 107 proteins were differentially abundant during the course of the training program. The differentially abundant proteins are presented here grouped by functional classification. In the research article associated with this data [1], the temporal changes in the cardiac proteome are discussed in the context of cardiac remodelling and development of a trained heart phenotype.

**Specifications Table**

**Table:** 

Subject area	*Biology*
More specific subject area	*Cardiac remodelling*
Type of data	*Table*
How data was acquired	*iTRAQ, LC-MS/MS*
Data format	*Globally normalized quantitation and analysis*
Experimental factors	*Total protein was extracted from ventricles then digested with trypsin; peptides were labeled with isobaric tags from 8-plex iTRAQ kits.*
Experimental features	*The proteome of the cardiac ventricle was quantified after 0, 4, 7, and 14 d of moderate exercise training.*
Data source location	
Data accessibility	*Data is available with this article.*

**Value of the data**

• This is the first description of the rainbow trout cardiac proteome. Rainbow trout are an important fish model species and are broadly used to study physiological responses to biotic and abiotic variables. Therefore, this data offers a benchmark for studying changes in the cardiac proteome associated with many relevant challenges including aquatic stressors associated with human-imposed environmental degradation, pollution, and climate change.
• This data describes protein abundance changes that occur in the fish heart during early stages of exercise training. Whether or not all of the observed changes accurately reflect effects at higher levels of biological organization (ex. enzyme activity, ATP production capacity) is an interesting avenue for further investigation.
• Comparing the temporal shifts in the trout cardiac proteome associated with exercise training with those of mammalian models can provide deeper insight into the cellular pathways governing adaptive and maladaptive cardiac remodelling.

## 1 Data

The data presented here includes the full list of proteins identified in the ventricles of juvenile rainbow trout hearts, and the quantitative changes to these proteins during exercise training. Mass spectra were searched against two separate databases, and the results of both searches are provided separately in alphabetical order of protein name and with corresponding accession numbers (Supplementary Table 1). The proteome was quantified in 3–4 biological replicates at each of 4 durations of exercise training: Day 0, Day 4, Day 7, and Day 14 of continuous swimming at moderate intensity. The average abundances of proteins at Day 0 can be sorted into numerical order to facilitate viewing of the most and least abundant proteins in a typical juvenile trout heart. Additionally, the relative abundance of each identified protein at Days 4, 7, and 14 were compared to control (Day 0) levels, and the complete list of differentially abundant proteins at each time point, grouped by function, is provided (Supplementary Table 2).

## 2 Experimental Design, Materials and Methods

### 2.1 Exercise training

Full methodological details are available in [1]. Briefly, juvenile rainbow trout (*O. mykiss*) of mixed sex and uniform size (n=27; 21.6±0.31 cm; 118.9±5.7 g) were exercised continuously at moderate intensity (approximately 2.0 BL s^−1^ on Day 1, and 1.7 BL s^−1^ on Day 14, based on average fork length) in a custom circular raceway. Water velocity was chosen to estimate 60% U_crit_ for rainbow trout of similar size [2], and to reflect previous studies where changes in relative ventricular mass [3] and cardiac function [4] were observed in trout swum at similar speeds for 4 wk. Fish were fed Profishent fish feed (Martin Mills Inc., Elmira ON), *ad libitum* once daily throughout the experimentation period. Prior to increasing water velocity (Day 0), and then on days 4, 7, and 14, a sub-sample of trout were euthanized in buffered MS-222 (50 mg L^−1^; n=6–7 per time point). The ventricle was removed and rinsed twice with saline buffer to clear luminal blood, then snap frozen on dry ice. Gender was assigned based on the colour of the developing gonads, with ovaries having a distinct orange hue relative to the pale pink testes; however, all fish were sexually immature. All protocols were approved by the University of Guelph Animal Care Committee (Protocol #2710), as per the guidelines of the Canadian Council for Animal Care.

### 2.2 iTRAQ Labeling

The ventricles of 2 male and 2 female fish per time point (except Day 7 which had 2 males and 1 female) were manually pulverized with a mortar and pestle, then homogenized in SDS buffer (4% w/v SDS, 100 mM HEPES, 0.1 M DTT, pH 7.6) containing 1x protease inhibitor (Roche, Mississauga, ON). The crude homogenate was clarified by centrifugation at 16 000 g for 10 min. Proteins from the supernatant were extracted using the Calbiochem Protein Extraction Kit (EMD Millipore, Billerica, MA), as instructed by the manufacturer. The protein pellet was dissolved in HEPES buffer (1 M HEPES, 8 M urea, 2 M thiourea, 4% CHAPS w/v; pH 8.5) and protein concentration determined using the Pierce BCA Protein Assay Kit (Thermo-Fisher, Whitby, ON). For each sample, 200 μg of protein was transferred to an Amicon® Ultra-0.5 centrifugation filter devices (10 K nominal molecular weight limit), in which samples were washed three times with UA buffer (8 M urea in 0.1 M HEPES, pH 8.5) before being incubated for 30 min in the dark with UA buffer containing 0.05 M of iodoacetamide (IAA, Sigma-Aldrich, Oakville, ON) and washing three times with 0.5 M of triethylammonium bicarbonate (TEAB, Sigma). Sequence-modified trypsin (Thermo-Fisher) was dissolved in 0.5 M TEAB and was added to each sample at a 1:50 enzyme to protein ratio. Samples were trypsin digested overnight (approximately 18 h) at 37 °C.

Digested peptides from the 4 biological replicates per time point were labelled using 2 8-plex iTRAQ kits (Thermo-Fisher), as outlined in the manufacturer׳s protocol. One Day 0 sample was labelled in duplicate reactions for inclusion as a standardization control across the 2 plexes. All experimental groups were represented within each 8-plex. Isobaric tags were alternated between experimental groups to avoid the unlikely potential for labelling bias between tags [5]. Following peptide labelling, samples within each 8-plex were pooled and purified through a C18 column (Sigma). Labelled peptides were eluted with 70% acetonitrile and 0.1% formic acid.

### 2.3 Mass spectrometry

Mass spectrometry was carried out at SickKids Proteomics Analysis Robotics & Chemical Biology Centre (SPARC BioCentre, Toronto, ON). Peptides were loaded onto a 50 cm x 75 μm ID column with RSLC 2 um C18 resin (EASY-Spray, Thermo-Fisher) with an integrated emitter. The Easy-Spray nLC 1000 chromatography system (Thermo-Fisher) was used to elute peptides onto a Q-Exactive hybrid mass spectrometer (Thermo-Fisher) using a solvent gradient (0 to 35% acetonitrile in 0.1% formic acid) over 4 h. The mass spectrometer was operated in the data dependent mode with 1 MS followed by 10 MS/MS spectra. Resolution of MS scans were either 70 000 (MS) or 17 500 (MS/MS) FWHM, with a target of 1×106 ions and maximum scan time of 120 ms. A relative collision energy of 27% was used for MS/MS. First mass was fixed at 80 Da with a dynamic exclusion of 15 seconds for MS/MS scans. Raw data files were acquired with XCaibur 2.2 and processed with Proteome Discover 1.4 (Thermo-Fisher).

### 2.4 Data Analysis

Identification and quantification of cardiac proteins was performed by PEAKS Studio 7 (Bioinformatics Solutions Inc., Waterloo, ON, Canada). The mass spectra were searched against the ray-finned fishes (*Actinopterygii*) NCBI non-redundant protein database and Uniprot/Swissprot database (taxonomy not selected) downloaded on June 16, 2015. Carbamidomethylation was set as a variable modification and iTRAQ as a fixed modification on N-termini and lysine residues. Search parameters allowed up to two missed trypsin cleavages, 10.0 ppm parent mass error tolerance and 0.02 Da fragment mass error tolerance. A high confidence in the peptide-protein identifications was assured by using a FDR cut-off of 1% and requiring a protein identification score (−10lgP) of ≥ 20 [6] and at least one unique peptide for protein identification [7], [8]. More than 99% of proteins had a protein identification score > 30 (equivalent P-value = 0.001), indicating these scores have less than 0.1% chance of being a random match, and all proteins included in the analysis were identified on both 8-plexes. An identical sample was included on each 8-plex to standardize abundance values between the two plexes. Once standardized, the two 8-plexes were combined for analysis, and only proteins that were quantified in all samples from both 8-plexes were considered for analysis. Statistical analysis of differentially abundant proteins was conducted using Linear Models for Microarray Analysis (LIMMA) implemented as a Bioconductor package in the R statistical interface [9]. Prior to statistical analysis, abundance values were globally normalized using variance stabilization in the R-based package ‘VSN’ [10]. Protein quantification using iTRAQ tends to underestimate fold changes compared to other approaches, therefore uncorrected P-values <0.05 were used to determine significant changes [11]. Proteins that were classified as unnamed proteins from the NCBI database were identified using Blastp, and then assigned the corresponding human ortholog protein accession ID.

## References

[1] Dindia LA, Alderman SA, Gillis TE. Novel insights into cardiac remodelling revealed by proteomic analysis of the trout heart during exercise training, *J Proteom* 161, 2017, 38-46. 10.1016/j.jprot.2017.03.023 28365405

[2] Richards J.G., Mercado A.J., Clayton C.A., Heigenhauser G.J.F., Wood C.M. (2002). Substrate utilization during graded aerobic exercise in rainbow trout. J Exp Biol.

[3] Greer Walker M., Emerson L. (1978). Sustained swimming speeds and myotomal muscle function in the trout, *Salmo gairdneri*. J Fish Biol.

[4] Farrell A.P., Johansen J.A., Suarez R.K. (1991). Effects of exercise training on cardiac performance and muscle enzymes in rainbow trout, *Oncorhynchus mykiss*. Fish Physiol Biochem.

[5] Burkhart J.M., Vaudel M., Zahedi R.P., Martens L., Sickmann A. (2011). iTRAQ protein quantification: A quality-controlled workflow. Proteomics..

[6] Zhang J., Xin L., Shan B., Chen W., Xie M., Yuen D., Zhang W., Zhang Z., Lajoie G.A., Ma B. (2012). PEAKS DB: De novo sequencing assisted database search for sensitive and accurate peptide identification. Mol Cell Proteomics.

[7] Gupta N., Pevzner P.A. (2009). False discovery rates of protein identifications: A strike against the two-peptide rule. J Proteome Res.

[8] Veenstra T.D., Conrads T.P., Issaq H.J. (2004). Commentary: what to do with “one-hit wonders”?. Electrophoresis..

[9] Ritchie M.E., Phipson B., Wu D., Hu Y., Law C.W., Shi W., Smyth G.K. (2015). Limma powers differential expression analyses for RNA-sequencing and microarray studies. Nucleic Acids Res.

[10] Huber W., von Heydebreck A., Sültmann H., Poustka A., Vingron M. (2002). Variance stabilization applied to microarray data calibration and to the quantification of differential expression. Bioinformatics..

[11] Mahoney D.W., Therneau T.M., Heppelmann C.J., Higgins L., Benson L.M., Zenka R.M., Jagtap P., Nelsestuen G.L., Bergen H.R., Oberg A.L. (2011). Relative quantification: characterisation of bias, variability and fold changes in mass spectrometry data from iTRAQ-labelled peptides. J Proteome Res.

